# Pregnant women and infants as sentinel populations to monitor prevalence of malaria: results of pilot study in Lake Zone of Tanzania

**DOI:** 10.1186/s12936-016-1441-0

**Published:** 2016-07-29

**Authors:** Ritha A. Willilo, Fabrizio Molteni, Renata Mandike, Frances E. Mugalura, Anold Mutafungwa, Adella Thadeo, Edwin Benedictor, Jessica M. Kafuko, Naomi Kaspar, Mahdi M. Ramsan, Osia Mwaipape, Peter D. McElroy, Julie Gutman, Rajeev Colaco, Richard Reithinger, Jeremiah M. Ngondi

**Affiliations:** 1 RTI International, Dar es Salaam, Tanzania; 2 National Malaria Control Programme, Dar es Salaam, Tanzania; 3 Swiss Tropical and Public Health Institute, Dar es Salaam, Tanzania; 4 Sengerema Health Institute, Sengerema, Tanzania; 5 President’s Malaria Initiative/United States Agency for International Development, Abuja, Nigeria; 6 President’s Malaria Initiative/United States Agency for International Development, Dar es Salaam, Tanzania; 7 President’s Malaria Initiative and Malaria Branch, Centers for Disease Control and Prevention, Atlanta, USA; 8 Malaria Branch, Division of Parasitic Diseases and Malaria, Centers for Disease Control and Prevention, Atlanta, GA USA; 9 RTI international, Washington DC, USA

**Keywords:** Malaria surveillance, Sentinel population, Pregnant women, Infants, Tanzania

## Abstract

**Background:**

As malaria control interventions are scaled-up, rational approaches are needed for monitoring impact over time. One proposed approach includes monitoring the prevalence of malaria infection among pregnant women and children at the time of routine preventive health facility (HF) visits. This pilot explored the feasibility and utility of tracking the prevalence of malaria infection in pregnant women attending their first antenatal care (ANC) visit and infants presenting at 9–12 months of age for measles vaccination.

**Methods:**

Pregnant women attending first ANC and infants nine to 12 months old presenting for measles vaccination at a non-probability sample of 54 HFs in Tanzania’s Lake Zone (Mara, Mwanza and Kagera Regions) were screened for malaria infection using a malaria rapid diagnostic test (RDT) from December 2012 to November 2013, regardless of symptoms. Participants who tested positive were treated for malaria per national guidelines. Data were collected monthly.

**Results:**

Overall 89.9 and 78.1 % of expected monthly reports on malaria infection prevalence were received for pregnant women and infants, respectively. Among 51,467 pregnant women and 35,155 infants attending routine preventive HF visits, 41.2 and 37.3 % were tested with RDT, respectively. Malaria infection prevalence was 12.8 % [95 % confidence interval (CI) 11.3–14.3] among pregnant women and 11.0 % (95 % CI 9.5–12.5) among infants, and varied by month. There was good correlation of the prevalence of malaria among pregnant women and infants at the HF level (Spearman rho = 0.6; p < 0.001). This approach is estimated to cost $1.28 for every person tested, with the RDT accounting for 72 % of the cost.

**Conclusions:**

Malaria infection was common and well correlated among pregnant women and infants attending routine health services. Routine screening of these readily accessible populations may offer a practical strategy for continuously tracking malaria trends, particularly seasonal variation. Positivity rates among afebrile individuals presenting for routine care offer an advantage as they are unaffected by the prevalence of other causes of febrile illness, which could influence positivity rates among febrile patients presenting to outpatient clinics. The data presented here suggest that in addition to contributing to clinical management, ongoing screening of pregnant women could be used for routine surveillance and detection of hotspots.

## Background

Populations living in sub-Saharan Africa are at greatest risk for malaria, where approximately 90 % of deaths are estimated to occur, with 70 % of deaths occurring in children under 5 years of age [1]. Tanzania has a substantial burden of malaria. Among children under-five, recent population-based, cross-sectional surveys in 2011/12 documented an overall national prevalence of malaria parasitaemia of 9.2 %, ranging from 0 to 31.8 % across the administrative regions [2]. Malaria parasitaemia is similarly prevalent among pregnant women, ranging from 5.5 to 11.5 % [3, 4].

Over the last decade, Tanzania has gradually scaled-up malaria control interventions. Insecticide-treated nets (ITNs) have been distributed through various campaigns, including: the Tanzania National Voucher Scheme introduced in 2004 targeting vulnerable groups, such as pregnant women and children aged under five [5]; the children under five catch-up campaign (U5CC) in 2009 [6]; and, the universal coverage campaign (UCC) in 2011 [7]. Artemisinin-based combination therapy (ACT) was introduced as first-line treatment in all public facilities in 2006 and malaria rapid diagnostic tests (RDTs) were introduced in 2009 [8]. Indoor residual spraying (IRS) was introduced in two districts of Kagera Region in 2007 and expanded to 18 districts in the Lake Zone by 2011 [8], where malaria prevalence measured by RDT in children aged 6–59 months was higher (30–41 %) than the rest of mainland Tanzania (18 %) in 2007–2008 [9]. Following the effective scale-up of multiple malaria control interventions, there has been a 45 % reduction in all-cause under-five mortality from 1999 to 2010 [8]. In addition, the Tanzania HIV and Malaria Indicator Survey (THMIS) 2011–12 found that malaria prevalence among children aged 6–59 months had declined to 9 % nationally, and 8, 19 and 26 % in Kagera, Mwanza and Mara Regions, respectively [2]. As malaria control interventions are scaled-up, rational approaches are needed for monitoring impact over time. The 2008–13 National Malaria Control Programme (NMCP) Monitoring and Evaluation Plan proposed testing of pregnant women and children under 5 years of age attending reproductive and child health (RCH) clinics as a suitable sentinel population for monitoring longitudinal malaria morbidity trends [10]. This study piloted an active screening approach to monitor malaria parasitaemia prevalence in two sentinel populations: (1) pregnant women attending their first ANC visit; and, (2) infants eligible for measles vaccination, at 9–12 months of age attending selected RCH clinics in Kagera, Mara and Mwanza Regions, as well as to assess the costs of implementing this strategy.

## Methods

### Study settings

**Fig. 1:**
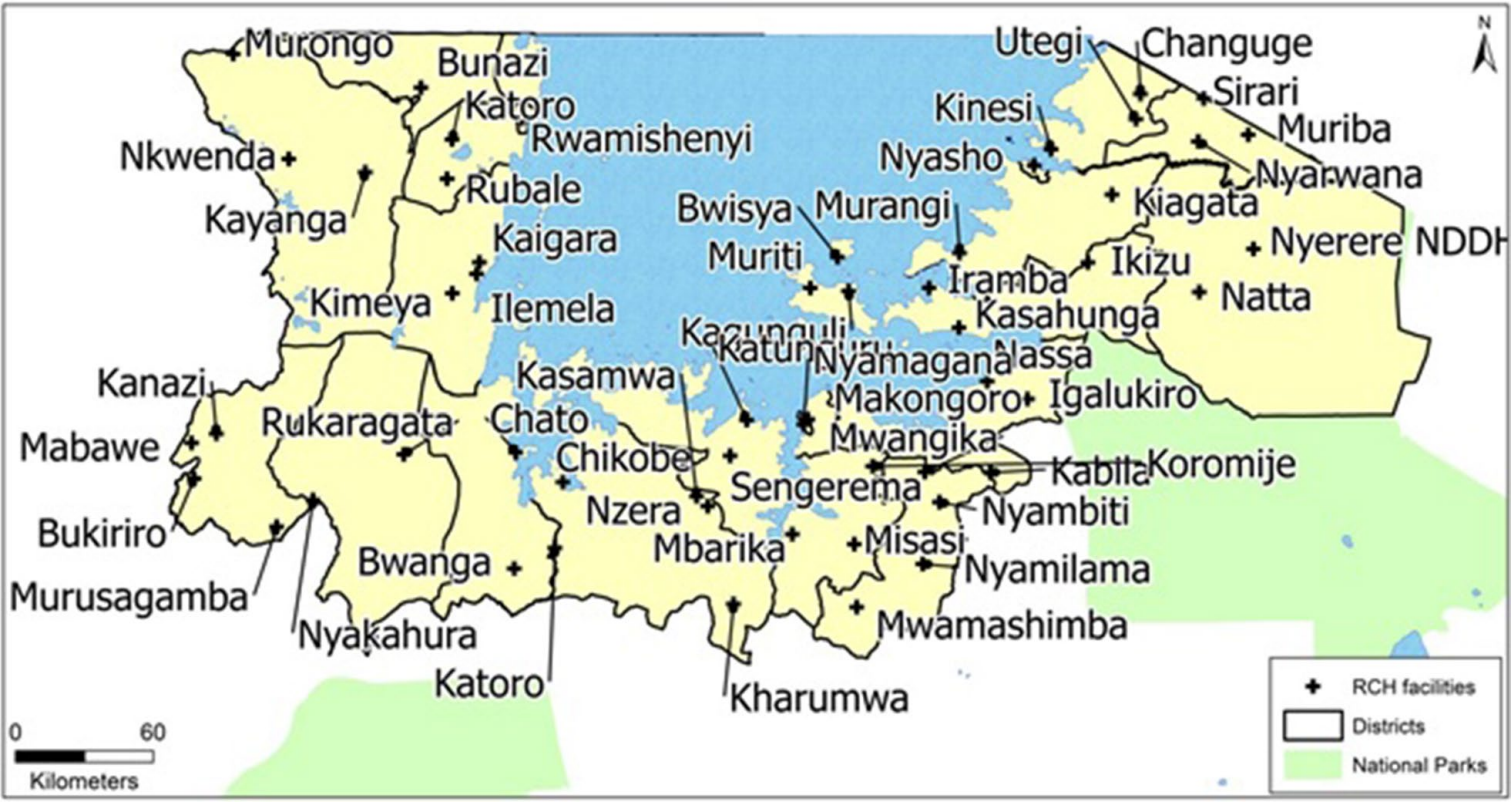
Geographic location of health facilities participating in the study, Mwanza, Mara and Kagera regions

The study was conducted from December 2012 to November 2013 in a non-probability sample of 54 health facilities where enhanced surveillance for fast tracking routine malaria data had been implemented and purposively sampled to provide geographical spread across the three regions. The health facilities included 49 (out of131 eligible) health centres (37.4 %) and five (out of 42 eligible) hospitals (11.9 %)) with dedicated RCH clinics in Kagera, Mwanza and Mara Regions in the Lake Zone of Tanzania (Fig. 1). This pilot was initially proposed in the 2008–13 National Malaria Control Program Monitoring and Evaluation Plan to inform future policy [10]. It was considered advantageous because: (1) there is high coverage in antenatal attendances and measles vaccination (over 90 %); (2) this population represents a homogeneous group than can be followed up longitudinally; (3) this population is easily reachable; (4) these data can provide prospective/longitudinal indications of malaria trends; (5) this system does not require extensive financial resources and is easily implementable under existing routine health care delivery systems; (6) low-levels of training are required; (7) data are easily recorded and reported using a modified information system; (9) there is the potential to add haemoglobin testing in the same facilities to monitor anaemia prevalence; and (10) this data collection system will provide a service to the target population as all positive cases will be treated immediately [10]. Subsequently, the 2014–2020 National Malaria Strategic Plan (MSP) endorsed the policy of screening women at first ANC [11], and in 2015, Tanzania started implementation of routine testing of pregnant women for malaria at first ANC as part of the antenatal profile. In addition, recording and reporting of ANC malaria screening results has been integrated into the routine health management information system (HMIS).

### Sample size estimation

Based on results of a malaria indicator survey done in 2011/12 [2], malaria prevalence in Kagera, Mwanza and Mara Regions was assumed to be approximately 5–15 %. A sample size of 300 people per group (women or infants) per month was chosen to be sufficient to allow estimation of prevalence of malaria of 15 %, with a 95 % confidence interval of 11.2–19.6 %. As prevalence decreases, if the number of women/infants screened remains constant, the confidence intervals around the estimates would widen.

### Malaria parasite detection

Health facility staff involved in the project were trained using the standard National Malaria Control Programme (NMCP) training manuals [12]. In participating RCH clinics, pregnant women attending their first antenatal clinic (ANC) visit and infants attending for measles vaccination were recruited to participate in testing for malaria infection with RDT. After obtaining consent, the RCH nurses obtained capillary blood via finger or heel (for infants) stick for malaria testing using RDT (Malaria Ag P.f/Pan, SD Bioline^®^). In addition to completing the routine RCH clinic register, test results were recorded in a study logbook. No data were collected on symptoms. Participants with positive RDT results were offered immediate treatment with either artemether-lumefantrine, for children and pregnant women in their second or third trimester, or quinine for pregnant women in their first trimester, according to national treatment guidelines.

Data on total number of attendees, total number tested with RDTs and total number of positive RDTs for both pregnant women and infants were aggregated at the facility level on a monthly basis and submitted to the study coordinator for analysis. The district malaria focal person was responsible for collecting and submitting the monthly data summary sheets from his/her respective district.

### Analysis of costs

The direct costs of implementing the RCH pilot were estimated. The direct cost elements included: training of RCH staff; RCH staff time; consumables (RDT and anti-malarials); and, travel expenses for monitoring and supervision. Training of RCH staff included cost of training venue, accommodation for RCH staff, printing of study guidelines and reporting forms. Using reported RCH attendance, it was estimated that on average two eligible infants and three eligible pregnant women attended RCH clinic daily. Assuming that each test takes approximately 20 min to perform and read [13], it was estimated that RCH nurses spent 2 h per day on RDT tests and recording of test results. The RDT was considered to be the only additional consumable for the testing, and included both purchasing costs ($0.55 average price for Pf/PAN, SD Bioline in 2013) [14] and distribution costs incurred by the Medical Stores Department (MSD) to supply to health facilities. The number of RDTs required was based on the reported RCH attendance for infants and pregnant women for the 12-month study period, assuming 100 % of attendees were tested. Finally, the travel cost for district staff to travel to health facilities for monitoring, supervision and collection of monthly data summaries was estimated. Costs were converted from Tanzania shilling (TZs) to US dollars (US$) based on the average exchange rate for 2013 of TZs 1600 per US$.

### Data analysis

Data were entered and cleaned in a Microsoft Excel spreadsheet. Data analysis was conducted using Stata 12.0 (Stata Corporation, College Station, TX, USA). Descriptive statistics were used to explore attendance at RCH clinics and proportion tested for malaria. Multilevel mixed-effects logistic regression models were fitted to adjust confidence intervals of prevalence estimates for clustering of data at health facility levels using the *melogit* routine in Stata [15]. Differences between proportions were compared using Chi squared tests. Spearman correlation was use to investigate association between proportion of participants tested and malaria positivity and correlation of malaria prevalence estimates between pregnant women and infants. To investigate geographic variation in attendance, malaria testing and malaria prevalence, raster maps were developed using inverse distance weighting (IDW) based on weighted combination of RCH facilities using ArcGIS software (ESRI, Redlands, CA, USA). This technique assumes that malaria data from a certain health facility are from people residing within a certain distance from that facility and therefore the variable being mapped (testing rate and malaria prevalence) decreased in accuracy with increasing distance from its sampled location [16]. Data were examined for spatial autocorrelation using Moran’s I test [17, 18].

## Results

### Reporting by health facilities

**Fig. 2:**
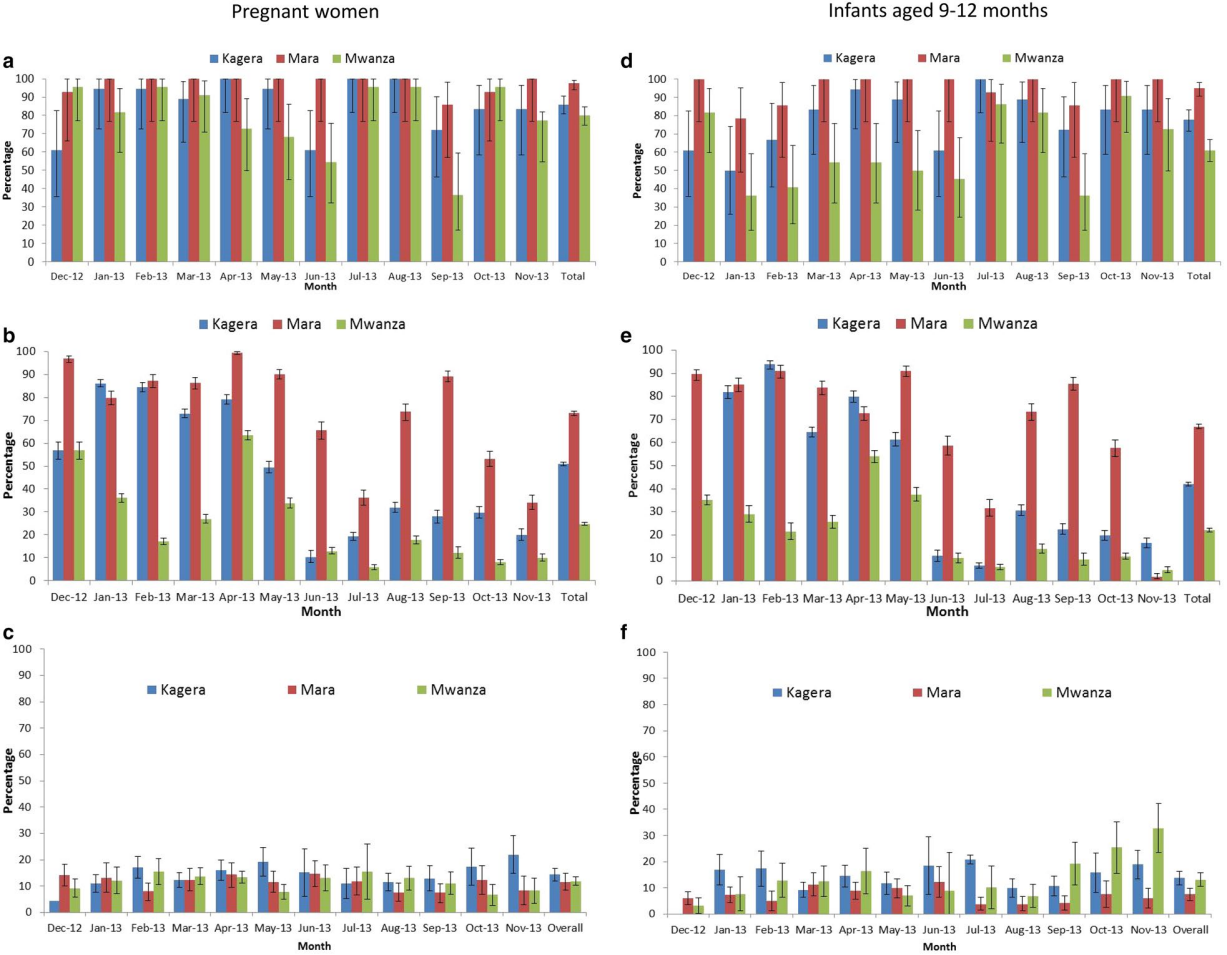
Distribution of reporting by RCH clinics, testing for malaria and malaria positivity among pregnant women and infants by region, December 2012–November 2013. Pregnant women: **a** proportion of RCH clinics reporting; **b** proportion tested for malaria; **c** proportion positive for malaria. Infants aged 9–12 months: **d** proportion of RCH clinics reporting; **e** proportion tested for malaria; **f** proportion positive for malaria

A total of 54 health facilities (31.2 % of eligible sites) were purposively sampled with geographical spread across the three regions. Of the monthly reports expected from the 54 health facilities participating in the pilot, 94.0 and 78.1 % were received for pregnant women and infants, respectively (Table 1). The median annual RCH attendance per health facility was 689 (range 107–4911) and 533 (range 69–1678) for pregnant women and infants, respectively. Mara Region had the most complete monthly reporting for both pregnant women (95.2 %) and infants (92.9 %), while Mwanza Region had the lowest reporting rates for both populations (85.6 % for pregnant women and 61.7 % for infants). Figure 2a, d show the trends of reporting by month.

**Table 1:** Summary of reporting by region, December 2012–November 2013

Characteristic	Region			
	Kagera	Mara	Mwanza	Total
Number of health facilities with RCH clinics	48	49	58	155
Number of health facilities participating: n (%)	18 (37.5)	14 (28.6)	22 (37.9)	54 (34.8)
Number of monthly health facility reports expected	216	168	264	648
Pregnant women				
Number of reports received	203	160	226	589
Proportion of reports received (%)	94.0	95.2	85.6	90.9
Infants aged 9–12 months				
Number of reports received	187	156	163	506
Proportion of reports received (%)	86.6	92.9	61.7	78.1

### RCH clinic attendance and malaria testing

**Fig. 3:**
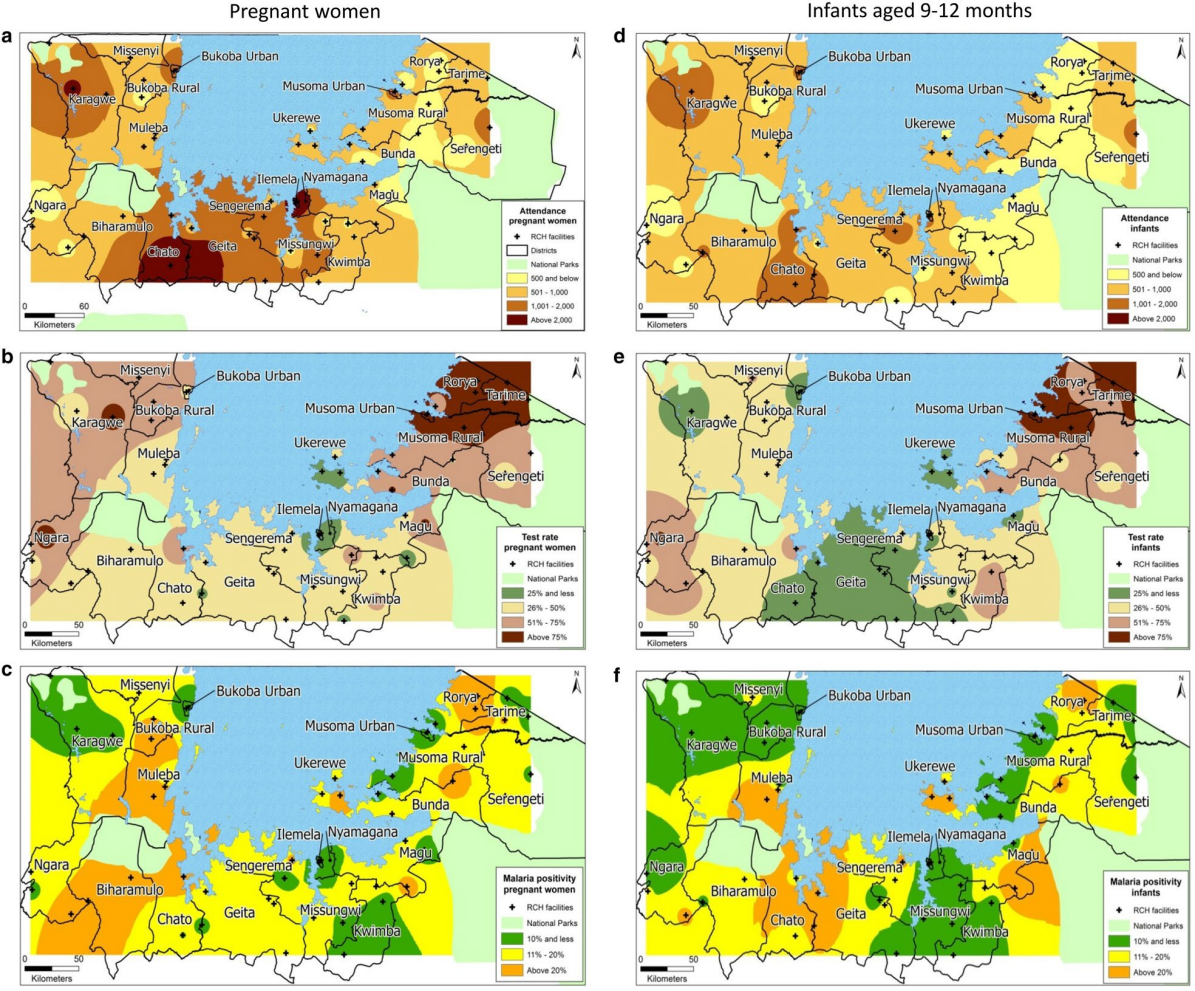
Geographical variation in RCH clinics attendance, malaria testing and malaria prevalence among pregnant women and infants by district. Pregnant women: **a** number attending RCH clinics; **b** proportion tested for malaria; **c** proportion positive for malaria. Infants aged 9–12 months: **d** number attending RCH clinics; **e** proportion tested for malaria; **f** proportion positive for malaria

A total of 51,467 pregnant women attended their first ANC visit and a total of 35,155 infants attended for measles vaccination at the 54 RCH clinics during this pilot. Overall, 21,184 (41.2 %) of pregnant women and 13,130 (37.3 %) of infants were screened for malaria infection (Table 2). Malaria screening rates varied markedly over the study period (Fig. 2b, e) and by location (Fig. 3b, e). Mwanza Region consistently had the lowest testing rates, while in Kagera testing was high in the first 4 months of the study, but declined markedly thereafter (Fig. 2b, e).

**Table 2:** Summary of attendance, testing and malaria positivity by region, December 2012–November 2013

Characteristic	Pregnant women				Infants aged 9–12 months			
	Kagera	Mara	Mwanza	Total	Kagera	Mara	Mwanza	Total
Number of participants attending clinic	16,844	9177	25,446	51,467	13,798	8050	13,307	35,155
Number tested	8283	6701	6200	21,184	4823	5619	2688	13,130
Proportion of participants tested (%)	49.2	73.0	24.4	41.2	35.0	69.8	20.2	37.3
Number positive	1158	782	733	2673	668	417	360	1445
Malaria positivity: % (95 % CI)	14.3 (11.9, 16.8)	11.5 (8.4, 14.7)	11.9 (10.2, 13.6)	12.8 (11.3, 14.3)	13.7 (11.1, 16.3)	7.5 (5.2, 9.8)	13.1 (10.5, 15.8)	11.0 (9.5, 12.5)

### Malaria prevalence

Among those tested, 12.8 % [95 % confidence interval (CI) 11.3–14.3] of pregnant women and 11.0 % (95 % CI 9.5–12.5) of infants were positive for malaria infection (Table 2). Malaria infection prevalence varied over the study period (Fig. 2c, f) with monthly prevalence of 9.9–14.6 and 5.2–16.7 % among pregnant women and infants, respectively, with marked variation by location (Fig. 3c, e). Malaria infection prevalence was highest in Kagera Region (14.3 and 13.7 % among pregnant women and infants, respectively) and lowest in Mara Region (11.5 and 7.5 % among pregnant women and infants, respectively).

### Variation of health facility attendance, malaria testing and malaria prevalence

Figure 3 shows the weighted average estimates of health facility attendance, malaria test rates and malaria prevalence among pregnant women and infants based on inverse distance weighing of the health facility observations. There was wide variation across the study areas in both the proportion tested (Fig. 3b, e) and proportion positive (Fig. 3c, f), with evidence of high malaria positivity (hotspots) in defined geographic locations. There was good correlation between prevalence of malaria among pregnant women and infants across the geographic distribution of reporting sites, especially in the high transmission areas (Fig. 3c, f). Moran’s I test showed that there was no spatial autocorrelation of health facility level malaria prevalence among pregnant women (p = 0.155) and infants (p = 0.353). The health facility prevalence of malaria in pregnant women was correlated with malaria prevalence in infants (Spearman rho = 0.6; p < 0.001). There was no correlation between malaria prevalence and proportion of participants tested for malaria among pregnant women (Spearman rho = −0.04; p = 0.8) or infants (Spearman rho = −0.2; p = 0.3).

### Comparison of prevalence with malaria positivity in outpatient department

**Fig. 4:**
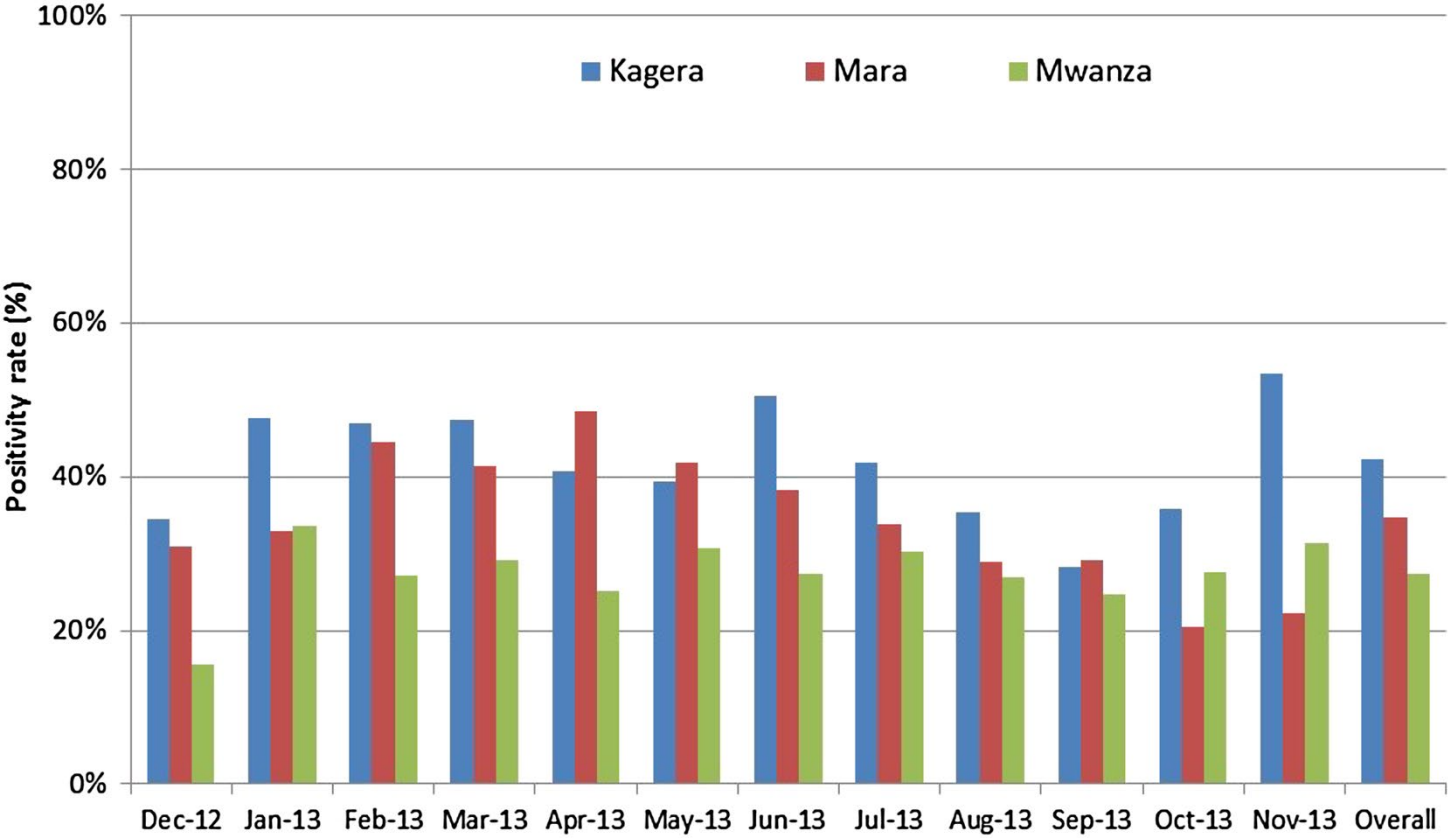
Malaria positivity among patients attending outpatient clinic in 54 health facilities participating in sentinel surveillance

**Fig. 5:**
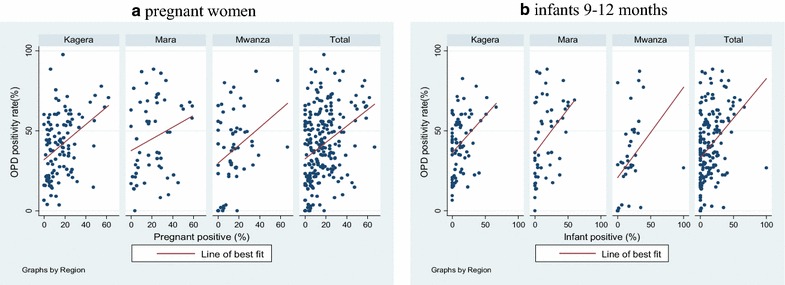
**a** Scatter plot of positivity rate at the outpatient department versus the prevalence among enrolled pregnant women. Spearman’s test: Kagera (rho 0.4, p < 0.001), Mara (rho = 0.2, p = 0.1), Mwanza (rho = 0.3, p = 0.36), Total (rho = 0.3, p < 0.001). **b** Scatter plot of positivity rate at the outpatient department versus the prevalence among enrolled infants. Spearman’s test: Kagera (rho = 0.4, p = 0.002), Mara (rho 0.5, p = 0.001), Mwanza (rho = 0.6, p < 0.001), Total (rho = 0.4, p < 0.001)

The malaria positivity rate in the outpatient department (OPD) of the participating facilities was collected in order to compare this to the prevalence among those attending RCH services. Overall, malaria positivity rates in the OPD varied by month and region, ranging from 15.6 to 53.0 %, with an average of 42.4, 34.8 and 27.4 % in Kagera, Mara and Mwanza regions, respectively (Fig. 4). Overall, there was a positive correlation between OPD positivity rate and positivity for both pregnant women and infants, although the correlation was only modest (Spearman’s rho = 0.3, p < 0.001for pregnant women and Spearman’s rho = 0.4, p < 0.001 for infants) (Fig. 5a, b).

### Costs analysis

The estimated direct costs of undertaking this study, based on RCH attendance, are summarized in Table 3. The average total cost of testing pregnant women and infants per health facility was estimated at US$ 2060.59 per year. The key cost driver was the cost of purchase and delivery of RDTs to the health facilities that accounted for 72 % of the total direct costs. It was estimated that this approach would cost US$1.28 for every person tested and the cost per positive test was estimated at US$ 10.70.

**Table 3:** Direct costs associated with the testing of pregnant women and children in RCH clinics

Cost elements	Annual cost (US$)		Proportion of total cost (%)
	Total for 54 health facilities	Average per health facility	
Training of RCH staff	7656.25	141.78	7
Personnel: RCH nurses	3240.00	60.00	3
Consumables (RDT)^a^	80,125.35	1483.80	72
Travel for monitoring and supervision	20,250.00	375.00	18
Total	111,271.60	2060.59	100

*RCH* reproductive and child health; *RDT* rapid diagnostic test

^a^Consumables based on reported attendance (assumes 100 % test rate for both pregnant women and infants). Cost of RDT based on average price for 2013 [14]

## Discussion

In an area of Tanzania with high malaria transmission where intensive vector control interventions (IRS and ITNs) were scaled up and complemented by universal access to ACT and RDT, nearly one in eight pregnant women and one in ten infants attending RCH clinics were positive for malaria infection. In general, the trends over time and space were similar for the two populations. Mapping the prevalence by location revealed the presence of localized high transmission hotspots for both the pregnant women and infants. While malaria test positivity rates from the OPD provide a source of data, which could be used to identify malaria hotspots, these data are influenced by other causes of fever. This study piloted a new approach for assessing population-based prevalence of malaria through targeted screening of readily accessible, asymptomatic populations (pregnant women and infants) that have high attendance at RCH clinics (95.9 % of pregnant women attend at least one ANC visit and 74.6 % of infants attend for measles vaccination) [19]. Compared to test positivity rate data obtained from the OPD, testing of a sentinel population (including both symptomatic and asymptomatic individuals) provides a more stable estimate over time, which is unaffected by other circulating illnesses. This data thus provides a better measure for monitoring disease trends over time. Cross-sectional household surveys are typically used to provide this data, however, these are costly and are performed at widely spaced intervals, which does not allow for fine scale tuning of intervention delivery in the interim. Ongoing, routine screening of readily accessible populations may offer a practical strategy for continuous monitoring to identify malaria hotspots and track the progress of malaria control over time. These data complement the data from the OPD, thus combining these sources could provide a more comprehensive understanding of the full epidemiologic picture.

In Tanzania, sentinel population testing of pregnant women and children under 5 years of age attending RCH clinics was initially proposed in the 2008–13 Malaria Monitoring and Evaluation Plan [10]. Following the pilot study, the 2014–2020 National Malaria Strategic Plan set a strategic intervention to establish countrywide longitudinal vigilance of malaria parasitaemia in sentinel populations: pregnant women and infants at RCH clinics, and school-age children [11]. While data obtained from these sentinel populations would not obviate the need for periodic cross-sectional population-based surveys to measure coverage of interventions, the available data suggest that the prevalence of anaemia and parasitaemia among children presenting to the health facility for routine immunization is correlated with that among children aged 6–30 months detected by household surveys [11]. Similarly, data from a recent meta-analysis by van Eijk found that the prevalence of malaria among pregnant women was strongly correlated with that from household surveys of children [20], suggesting that either of these populations could be used to provide ongoing information about community level prevalence. From a biological standpoint, monitoring of infants may be preferable to monitoring adult women. The prevalence among infants nine to 12 months of age should closely mirror that of all children 6–59 months. Since infants have not yet developed significant immunity, these infections are likely to have been recently acquired. Furthermore, infants are less likely to have travelled than pregnant women. Thus, the prevalence of infection among infants may be more likely to represent recent and local transmission dynamics than that in adult women. However, pregnant women are more likely to be infected with malaria than non-pregnant women, and are at greater risk for severe disease than non-pregnant women [21]. This is particularly true for primigravid women, who are at increased risk of severe disease, with higher risk of severe anaemia and maternal death, higher rates of miscarriage, intrauterine demise, premature delivery, low-birth-weight neonates, and neonatal death compared to multigravid women [22]. Identifying and treating infected pregnant women with asymptomatic infections who may otherwise have gone untreated may have the added benefit of improving birth outcomes [23]. In addition, this may have a benefit on transmission, as it has been suggested that pregnant women may be a reservoir of transmission [24]. Finally, during the first ante-natal care visit, a panel of blood tests is already performed, whereas children presenting for measles immunization would not routinely have a blood draw. For these reasons, in Tanzania, testing of pregnant women was felt to be a better choice for routine monitoring than infants, and in 2015, Tanzania started implementation of routine testing of pregnant women for malaria at first ANC as part of the antenatal profile [25]. In addition, recording and reporting of ANC malaria screening results has been integrated into the routine health management information system (HMIS).

The study estimated the direct costs associated with the sentinel population screening pilot. The costs included training, staff time required to perform and record the test, test kits, and travel expenses for monitoring and supervision. These estimates suggest that nearly three-quarters of direct costs were attributable to purchase and delivery costs for RDTs. Nonetheless, reporting of ANC malaria testing through routine HMIS, and integration of RDT quality assurance monitoring and supervision with other routine supervisory activities, that are undertaken on a quarterly basis by the district teams, would reduce these costs.

This study has a number of potential limitations. The low rate of testing in Kagera and Mwanza is a potential source of bias and may have underestimated the results. The low testing rates were largely attributed to stock-outs of RDTs during the study period. Several reasons were reported by RCH clinics to explain frequent RDT stock-outs, including: weak quantification and forecasting by health facilities, delayed delivery of RDTs to health facilities by MSD, and stock out at MSD central stores. The analysis suggests that malaria prevalence was not correlated with the proportion of participants tested; thus the low testing rate is not expected to have biased the results. Lack of reporting was partly related to testing activity because RCH clinics did not submit any reports where testing was absent due to RDT stock-outs. It is likely that the low reporting rates, especially among infants in Mwanza Region, may have biased the results. In this study, participating health facilities were purposively selected to provide a geographically spread sample, but were not probabilistically selected to support parametric statistics and generalizable estimates. While this may be a potential source of bias, sampling is not likely to have influenced reporting rates and RDT stock-outs. Finally, history of recent symptoms for the persons tested was not recorded, as it was not part of the routine ANC register, making it impossible to know if those who tested positive had fever or other malaria symptoms in recent days (but were asymptomatic at time of the RCH visit). Finally, there is evidence to suggest that the HRP-2/pLDH RDTs are not sufficiently sensitive to diagnose very low density malaria infections in asymptomatic pregnant women [26], thus some infections may be missed. Nonetheless, if this screening activity also worked to detect very early cases of uncomplicated malaria, then this approach could benefit low transmission areas where additional efforts are needed to reinforce passive case detection.

## Conclusions

Routine screening of pregnant women at first ANC and infants presenting for measles vaccination may offer a practical approach for ongoing monitoring to track the progress of malaria control over time. This strategy provides benefit to the tested individual by identifying and treating those with asymptomatic but detectable parasitaemia. In addition, the continuous data generated down to the sub-district level allows for targeting malaria control interventions according to the level of transmission. Of the two groups, testing of pregnant women is likely to be less disruptive to clinic flow, as pregnant women already have blood drawn for routine ANC screening, but infants may provide a more precise estimate of transmission variability across sites and over time. Malaria screening of pregnant women at first ANC should be considered as a practical method for continuous routine surveillance of malaria prevalence at the sub-district level.

## Abbreviations

ANC: antenatal clinic
ACT: artemisinin combination therapy
HMIS: health management information system
IDW: inverse distance weighting
IRS: indoor residual spraying
ITN: insecticide-treated net
LLIN: long-lasting insecticidal nets
RDT: rapid diagnostic test
MSD: medical stores department
NMCP: National Malaria Control Programme
OPD: outpatient department
RCH: reproductive and child health
THMIS: Tanzania HIV and Malaria Indicator Survey
WHO: World Health Organization
